# Niche Filling Dynamics of Ragweed (*Ambrosia artemisiifolia* L.) during Global Invasion

**DOI:** 10.3390/plants12061313

**Published:** 2023-03-14

**Authors:** Xing-Jiang Song, Gang Liu, Zeng-Qiang Qian, Zhi-Hong Zhu

**Affiliations:** 1 College of Life Sciences, Shaanxi Normal University, Xi’an 710119, China; 2 Research Center for UAV Remote Sensing, Shaanxi Normal University, Xi’an 710119, China; 3 Changqing Teaching & Research Base of Ecology, Shaanxi Normal University, Xi’an 710119, China

**Keywords:** niche shift, niche conservatism, invasive plant, invasion stage, ragweed

## Abstract

Determining whether the climatic ecological niche of an invasive alien plant is similar to that of the niche occupied by its native population (ecological niche conservatism) is essential for predicting the plant invasion process. Ragweed (*Ambrosia artemisiifolia* L.) usually poses serious threats to human health, agriculture, and ecosystems within its newly occupied range. We calculated the overlap, stability, unfilling, and expansion of ragweed’s climatic ecological niche using principal component analysis and performed ecological niche hypothesis testing. The current and potential distribution of *A. artemisiifolia* was mapped by ecological niche models to identify areas in China with the highest potential risk of *A. artemisiifolia* invasion. The high ecological niche stability indicates that *A. artemisiifolia* is ecologically conservative during the invasion. Ecological niche expansion (expansion = 0.407) occurred only in South America. In addition, the difference between the climatic and native niches of the invasive populations is mainly the result of unpopulated niches. The ecological niche model suggests that southwest China, which has not been invaded by *A. artemisiifolia*, faces an elevated risk of invasion. Although *A. artemisiifolia* occupies a climatic niche distinct from native populations, the climatic niche of the invasive population is only a subset of the native niche. The difference in climatic conditions is the main factor leading to the ecological niche expansion of *A. artemisiifolia* during the invasion. Additionally, human activities play a substantial role in the expansion of *A. artemisiifolia*. Alterations in the *A. artemisiifolia* niche would help explain why this species is so invasive in China.

## 1. Introduction

The rapid growth of global trade and increase in human travel has led to a rise in invasions by non-native species [1,2]. These invasive species not only cause harm to agriculture, economies, and ecosystems but also pose a threat to human health [3,4,5]. Invasive species have a strong ability to spread under different environmental conditions, and that spread may threaten biodiversity, lead to the extinction of local species, and drastically change local ecosystems [6,7,8,9]. When invasive species are introduced to a new area, their niche may change due to a lack of natural enemies or due to adaptations motivated by local conditions [10,11,12]. Therefore, niche shift analysis is an effective way to understand the determinants of species distributions and predict the expansion potential of invasive species [13,14].

The niche conservation of invasive species between native and non-native populations is a key factor in predicting the likelihood and risk of invasion [15,16,17]. However, there may be differences between the native and non-native niches. The tolerance of a species to environmental conditions changes rapidly due to novel selection pressures within its invasive range [15,18]. A niche shift occurs when there is niche space in the area of range expansion that is not occupied in the native range [18]. For example, factors such as predation, competition, and dispersal restrictions allow species to extend their range into areas that would have been inaccessible in their native environment [19]. Some studies have shown that niche conservation is common in invasive species [18,20,21,22,23]. However, a growing number of studies have shown that the native and non-native niches of an invasive species are not identical [15,18,19,24,25,26,27,28,29,30,31].

Niche mismatches between invasive and native areas are not due entirely to niche transfer. This misleading signal occurs when an invasive species occupies a niche space within its range that reflects only part of its native range [21]. For example, in the initial stage of a biological invasion, invasive organisms do not have the opportunity to spread and occupy all viable niches within the invaded area [18,32]. In addition, invasive organisms may experience a bottleneck effect during the invasion process, which can lead to a reduction of the ecological flexibility of that species within this new range of invasion [18]. To avoid the false signal of niche shift and better describe the post-invasion niche dynamics, a “COUE” (centroid shift, niche overlap, unfilled, extended) framework is suggested for use in describing the niche shift between native and invasive ranges [21,33]. The niche shift can be reflected as follows: (1) niche stability refers to the proportion of niche space existing in both native and invasive areas (i.e., niche conservation), (2) niche expansion is the proportion of the niche space existing only in the invasive range relative to the native range, and (3) niche unfilling is the proportion of the niche space existing only in the original range [21].

*Ambrosia artemisiifolia* L. is an annual herb native to North America. It was introduced to France in 1863 [34] and recorded in Queensland in 1930 [35] and China in 1935 [36]. *Ambrosia artemisiifolia* is extremely reproductive, producing 3000–6000 seeds per plant (up to 60,000). The seeds can retain germination potential up to and exceeding 40 years of dormancy in the ground [37]. In China, Europe, and North America, the highly allergenic nature of A. artemisiifolia pollen has seriously endangered human health and is one of the main causes of hay fever allergies [38]. *Ambrosia artemisiifolia*, which has strong allelochemical effects, usually competes with crops for light and nutrients, resulting in reduced crop yields [39]. It has been identified as one of the most dangerous invasive species in China [40].

Niche shifts are very important for revealing the invasive processes of non-native plants. Understanding how niches change during the invasive process will provide a more sufficient basis for risk evaluation and for the control of invasive plants [19]. In this study, we attempted to determine: (1) whether invasive populations of *A. artemisiifolia* exhibit ecological niche conservatism or ecological niche shift; (2) how its invasive niche has changed in different regions of the world compared to its native niche; and (3) whether it has reached an invasive equilibrium stage in China. Our study would help to understand the ecological niche changes of *A. artemisiifolia* during the invasion process as well as its invasive situation in China, which is critical for understanding the spread and distribution of *A. artemisiifolia* and developing effective management and control strategies.

## 2. Results

### 2.1. Niche Shift of A. artemisiifolia in Its Native and Invasive Range

**Figure 1:**
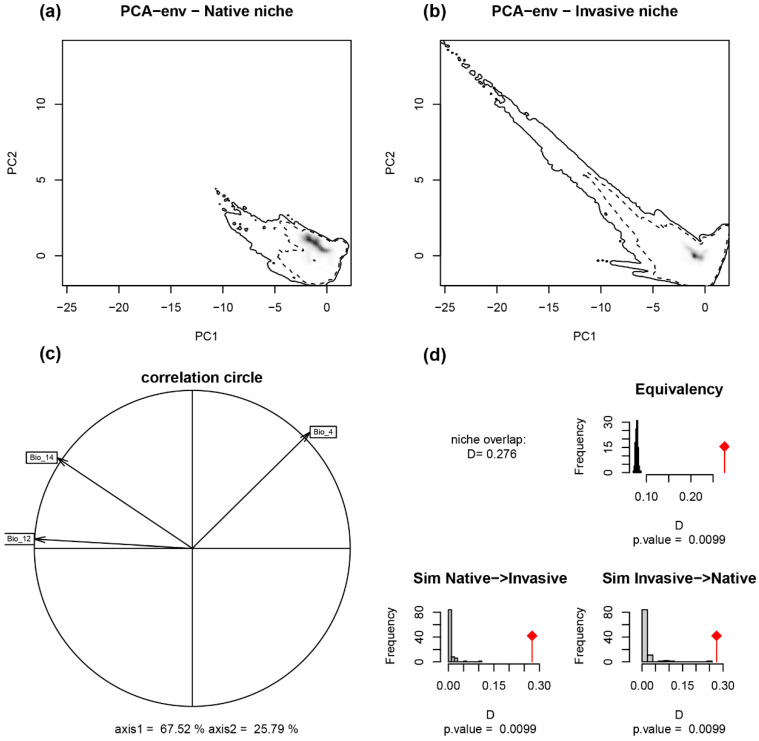
The native and all the invasive niches of *Ambrosia artemisiifolia* under the native climate conditions. Comparison of the ambient environmental conditions in native and invasive regions using a PCA calibrated by the environmental background (PCA-env) of the smoothed density of occurrences (gray shading) of (**a**) native and (**b**) all invasive sites. (**c**) The contribution of each variable to the principal component axes. (**d**) Representative histograms depicting the niche equivalence test and niche similarity test of the two regions.

Based on the characteristic analysis of the climate niche of the native and global invasion ranges, the first PCA axis (PC1: 67.52%) was closely related to precipitation, while the second principal component analysis axis (PC2: 25.79%) was related to temperature seasonality (Figure 1c). The climatic niches in the native range and the range of all global invasions overlap slightly (Schoener’s D = 0.276). The tests of niche equivalence and similarity showed that the observed value of niche overlap was always significantly larger than that of random niche overlap (*p* < 0.05) (Figure 1d). This indicates that the climatic niches in the ranges of native and all invaded areas are generally similar, though not equivalent.

The test of niche equivalence between the climate niche in all invasion ranges and that in the native range of *A. artemisiifolia* shows that they are equivalent nowhere other than Australia (Table 1). The results of the niche similarity test showed that the climate niche similarity between the native and invasion ranges was significantly higher than expected, except for in Africa. However, the climate niche space shared between the native range and Africa had greater similarity in that one direction (native and Africa) than would be expected by chance, indicating that the climate niche of the native range had more in common with that of Africa’s range than could be expected by chance; however, the reverse case was not observed (Table 1).

**Figure 2:**
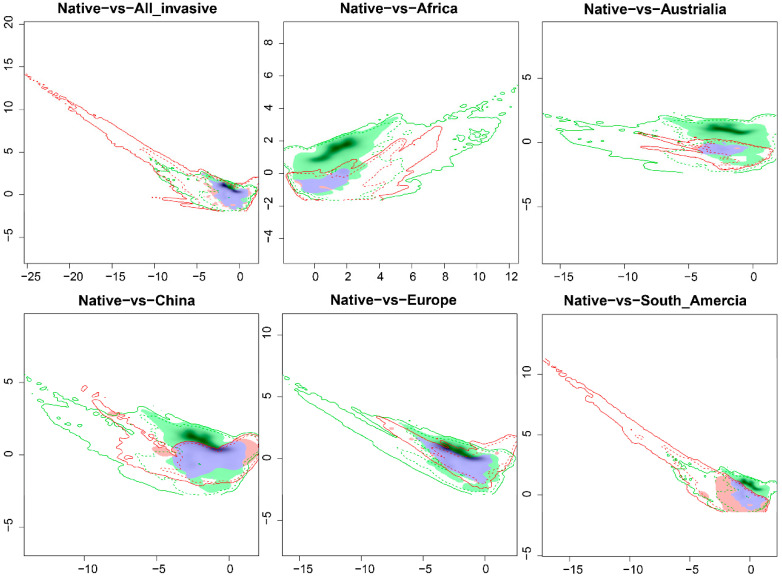
Projection of the native (green) and invasive regions (red) realized niches of *Ambrosia artemisiifolia* in climatic space, represented by the first two axes of principal component analysis. Contours represent 100% (solid line) and 50% (dotted line) of the available climatic space within each range (green native, red invasive). Unfilled, stable, and expanded niches are represented by green, blue, and red shades, respectively. The gray shading shows the smoothed occurrence density in the native niche space and in the exotic niche space.

The niche dynamic index showed that *A. artemisiifolia* was stable in the global invasion range, with only a large expansion in South America (Table 1). With the exception of South America, the climatic niches of the other invasive regions are almost a subset of the native North American niches (Figure 2). Among all the invasive regions in the world, the climate niche of South America is very unique, not only with a low Schoener’s D value but also with a highly unstable climate niche (Unfilling = 0.846, Expansion = 0.407) (Table 1). The climatic niche of South America and the native niche of North America expanded greatly along both axes of the PCA (Figure 2). In other invasive regions, *A. artemisiifolia* climatic niches were highly stable, especially in Europe. The climate niche of *A. artemisiifolia* in Europe was completely stable, with moderate niche overlap and unfilling (Table 1).

### 2.2. Predicted Potential Distribution of A. artemisiifolia in China

We filtered out the four factors that had the greatest influence on the current distribution of ragweed, temperature seasonality (BIO_4), annual precipitation (BIO_12), precipitation of driest month (BIO_14), and human influence index (HII), after eliminating a large number of factors with low contributions in pre-modeling. The HII was the most important factor in all three models we built (Table 2). Similarly, the contribution of the HII was highest in these models, with the only exception of the Native model, in which BIO_14 made the highest contribution (Table 2). This suggests that human activity is the most important factor influencing ragweed’s current global distribution.

**Figure 3:**
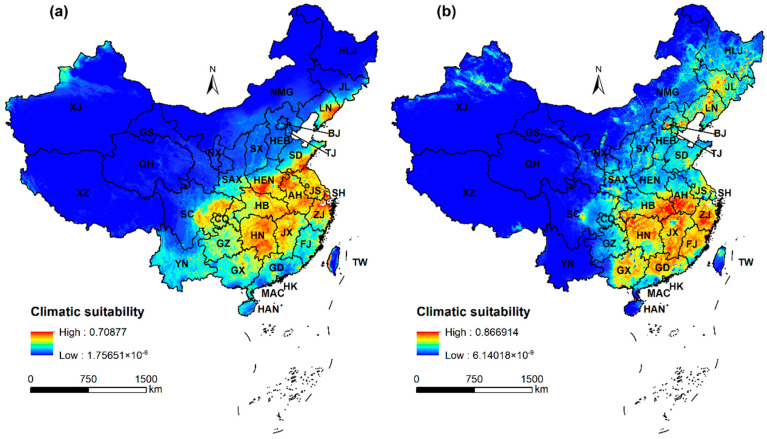
Prediction of the potential distribution of *Ambrosia artemisiifolia* in China based on occurrences from (**a**) native range (Native) and (**b**) China (Inv-China). Province abbreviations: AH = Anhui, BJ = Beijing, CQ = Chongqing, FJ = Fujian, GD = Guangdong, GS = Gansu, GX = Guangxi, GZ = Guizhou, HEN = Henan, HB = Hubei, HEB = Hebei, HAN = Hainan, HK = Hong Kong, HLJ = Heilongjiang, HN = Hunan, JL = Jilin, JS = Jiangsu, JX = Jiangxi, LN = Liaoning, MAC = Macau, NMG = Inner Mongolia, NX = Ningxia, QH = Qinghai, SC = Sichuan, SD = Shandong, SH = Shanghai, SAX = Shaanxi, SX = Shanxi, TJ = Tianjin, TW = Taiwan, XJ = Xinjiang, XZ = Tibet, YN = Yunnan, ZJ = Zhejiang.

**Figure 5:**
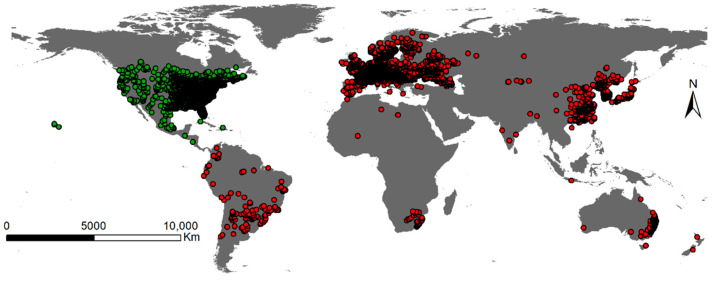
Records of native (green dots) and invasive (red dots) occurrences of *Ambrosia artemisiifolia* worldwide. The records of the occurrence of *A. artemisiifolia* were obtained from the Global Biodiversity Information Facility (GBIF) and the Chinese Virtual Herbarium (CVH).

The predicted potential distribution of *A. artemisiifolia* closely matches the current occurrence record (Figure 3 vs. Figure 5). The results of the Native and Inv-China models predicted that the areas with high suitability for *A. artemisiifolia* were highly similar, and they were all concentrated in east and central China (Figure 3). In addition, the results of the Native model (Figure 3a) show that there are only small areas of high suitability in Liaoning Province, except where those areas are concentrated. The results of the Inv-China model (Figure 3b) show that there are discrete areas of high suitability dispersed throughout northern China except where those areas are concentrated. Compared to the prediction results of the Native model (Figure 3a), the prediction results of the Inv-China model (Figure 3b) show that there are a large number of regions with low to medium suitability in northern China.

### 2.3. Invasion Stage Analysis

**Figure 4:**
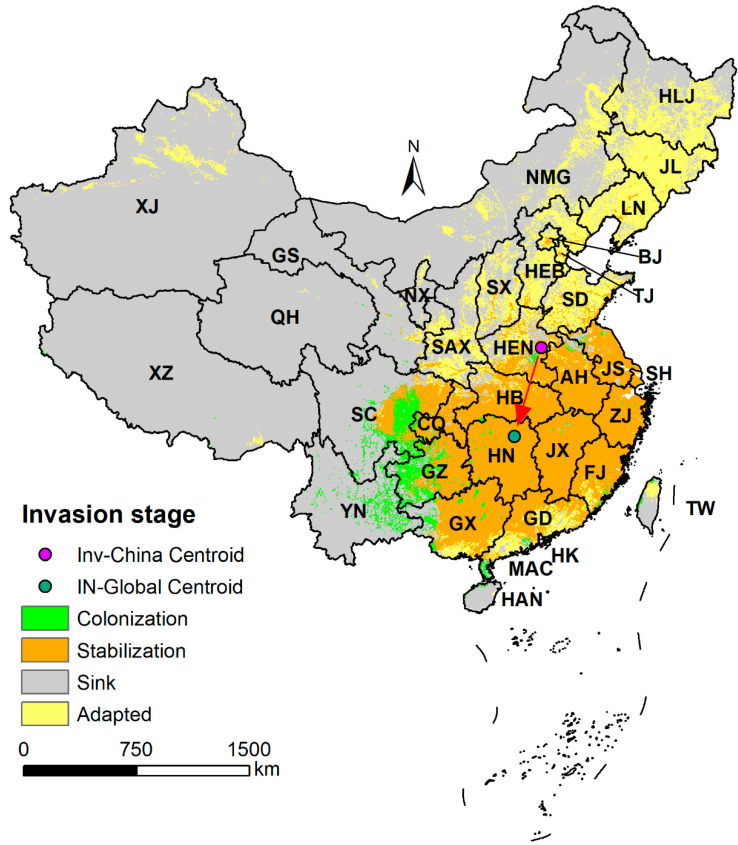
Observed occurrences of *Ambrosia artemisiifolia* at different invasion stages based on global (IN-Global) and regional (Inv-China) model predictions and mapped areas showing the potential (hypothesized) for population stabilization, adaptation, colonization, sink, and centroid transfer. The abbreviated name of each province is the same as in Figure 3.

Analysis of the *A. artemisiifolia* invasion stages based on regional (Inv-China) and global (IN-Global) models showed that the stable populations of *A. artemisiifolia* in China were mainly concentrated in central, east, and south China and some areas of Sichuan, Chongqing, and Guizhou in southwest China. Regional adaptations mainly occurred in the northern provinces of China and the southern periphery of the stable region. These areas are dominated by regional adaptation, with stable populations interspersed across individual provinces. Colonization occurred mainly in the periphery of stable regions such as Sichuan, Guizhou, Yunnan, and Guangxi in southwest China (Figure 4). The results showed that *A. artemisiifolia* had not reached equilibrium in the Chinese regional environment. By comparing the centroid results of the global model (IN-Global) and the regional model (Inv-China), we found that the centroid of the global model (IN-Global) clearly shifted southward relative to the centroid of the regional model (Inv-China) (Figure 4, red arrow).

## 3. Discussion

Niche conservatism is an expected part of a biological invasion [19,41]. However, the characteristics of niche shifts caused by organisms invading new ecosystems are often ignored. The niche shift during the biological invasion process may reflect the historical and/or biological processes occurring in the original range and the invasion range [19].

When Chapman et al. used a mechanistic model to study the niche of ragweed in Europe, they found that the niche of ragweed in Europe changed [42]. However, in this study, we found that *A. artemisiifolia* retained the climatic niche characteristics of its native range in most of the invasion areas; this was consistent with the expected hypothesis of niche conservatism. This result is consistent with the results of Petitpierre et al. on the climatic niche changes of *A. artemisiifolia* between its native region and Europe, which emphasizes the important role of niche conservation in the process of biological invasion [21]. Using occurrence data from North America, Cunze et al. accurately predicted the distribution of ragweed in Europe [43]. This phenomenon also suggests that ragweed maintained its niche during its invasion of Europe.

However, the niche instability of *A. artemisiifolia* was also detected in some of the invaded ranges. For example, its climatic niche changed sharply in South America and expanded greatly compared to that in the native range, showing characteristics of niche shift. Existing studies have shown that invasive species can quickly adapt to new conditions when they colonize new ranges [44,45]. The substantial seed yield of ragweed and the ability of the seeds to remain dormant in the soil for long periods provide the basis for its adaptation to new climates [37]. *Ambrosia artemisiifolia* occupies the largest area in the PCA space of South America (Figure 2), and most of the native climatic space is a subset of the climate space of South America, indicating that *A. artemisiifolia* has experienced a wide range of climate conditions in South America. It has been suggested that the observed niche shifts between native and invasive areas can be explained by changes in climatic conditions between the two areas [46]. Differences in background climate conditions may be the main driving force of the *A. artemisiifolia* niche shift in South America. The results of the niche similarity test also indicate that there are differences in the environmental space between South America and North America. Multivariate environmental similarity surface (MESS) analysis on the native range revealed that only a small portion of the climate in the South American range, located in the northwest corner of the continent, is similar to that in the native range of *A. artemisiifolia* (Supplementary Material Figure S1). In South America, climate conditions similar to those in *A. artemisiifolia’s* native range are extremely rare. The climate conditions in South America and North America are considerably different (Supplementary Material Figure S1). Invasive populations from South America thus cannot fill the climate space of the North American range [33]. Moreover, the *A. artemisiifolia* has expanded into new climates in the invasive range that are not found in the native range (Figure 5 vs. Supplementary Material Figure S1).

The niche shifts of *A. artemisiifolia* between its native range (North America) and Africa, Australia, China, and Europe were mainly due to the unfilled niche and the overall climate of the invasion area, which was a subset of the native climate space (Figure 2). Studies have found that unfilled niches are more common than niche expansion among non-native plants globally [21] and in Holarctic vertebrates [47,48]. This implies that suitable climatic conditions exist within the unoccupied invaded range, which we believe is due to ragweed failing to reach quasi-equilibrium within the invaded range [33]. A large number of unfilled niche spaces within these expansions are associated with non-climatic constraints, such as diffusion restrictions or effective control and management measures in the early stages of invasion [49,50,51,52]. In addition, the differences in unfilled spaces between different invasive areas may also be caused by genetic bottlenecks after the introduction of *A. artemisiifolia.* For example, the genetic diversity of non-European *A. artemisiifolia* populations is significantly lower than that of the native populations, while the genetic diversity of European populations is not significantly different from that of the native populations, suggesting that the introduced populations encountered different genetic bottleneck effects [53].

The species density of invasive populations in regions other than Europe outperformed those of native ragweed populations on the PC1 axis (precipitation), implying that ragweed does not fully occupy the climatic conditions of its native range. The presence of large numbers of unoccupied niche spaces in the native range may be due to pressures from natural enemies, such as insects, herbivores, or pathogens [54]. A study showed 450 species of insects, mites, and fungi associated with *Ambrosia spp.* in North and South America [49]. For example, infection of *A. artemisiifolia* by the fungus *Protomyces gravidus* in the United States and Canada can lead to stem gall disease and death [55]. The white rust fungus *Albugo tragopogi* in Canada can significantly reduce pollen and seed production of *A. artemisiifolia* [56]. In the U.S., Canada, and parts of Europe, the growth of *A. artemisiifolia* can also be suppressed by the ragweed beetle *Ophraella communa* [36,57]. Conversely, the lack of natural enemies of ragweed when it was first introduced facilitated its expansion in analog climates.

The climatic niche of all invaded areas was much cooler than that occupied by *A. artemisiifolia* in its native range (observed on the second PCA axis, Figure 2). This is consistent with the result of a previous study in which *A. artemisiifolia* differed in cold tolerance in its native and invasive ranges [58]. For instance, Gallien et al. speculate that *A. artemisiifolia* may have been shifting its niche toward more demanding alpine conditions over the past few decades [59]. This may be a result of local adaptation that occurs after introduction. An empirical study revealed that *A. artemisiifolia* adapted to the climate of invasion sites and expanded its invasion range through differentiated life-history traits [60]. For example, *A. artemisiifolia* increases the length of its reproductive period by flowering earlier in order to adapt to colder conditions at higher latitudes, thus producing more seeds [60]. Additionally, the considerable germination rate of *A. artemisiifolia* under highly variable environmental conditions is one of the factors of its success in achieving large-scale invasion [40].

The comparison results of the Native model and the IN-Global model with the Inv-China model both indicate that there are some unfilled areas in China (Figure 2 and Figure 3). This result fully shows that the invasive population of *A. artemisiifolia* in China has not yet reached a regional equilibrium and that China is still facing a high risk of invasion by *A. artemisiifolia*. Some studies have shown that invasive ragweed populations in China have been introduced multiple times, and the number of alleles is close to the population of origin [61,62]. Our results also show that the unfilled niche of ragweed in China is mainly caused by the colonization time lag. Compared to the centroid of the regional model, the centroid of the global model clearly shifts southward. This is primarily because the population of *A. artemisiifolia* in China mainly spreads from north to south [63]. This also suggests that *A. artemisiifolia* populations in China may have the potential to be more drought tolerant than those in other regions. Southwest China (Sichuan, Guizhou, Guangxi, Yunnan), which has not been invaded at present, faces a relatively high risk of invasion (Figure 3 and Supplementary Material Figure S2). This outcome is in line with the earlier findings made when *A. artemisiifolia*’s invasion risk in China was predicted using Maxent [64,65] and GLM [66].

The limited natural dispersal ability of ragweed and its highly segregated distribution in Xinjiang and Tibet (where the introduction of ‘spotfire’ led to a human-mediated jump dispersal) suggest that dispersal limitation is the main cause of its highly segregated distribution [67,68]. The provinces of Jiangsu, Shandong, Anhui, and Henan predicted by the Native model are highly suitable areas for *A. artemisiifolia*. However, these areas are moderately or even poorly suitable, as predicted by the Inv-China model. One of the reasons for this phenomenon may be due to the presence of the *A. artemisiifolia* beetle *O. community*, which is a natural foe used for biocontrol [51].

Biological invasion is a complex process of multifactor interactions [69]. The invasion process is driven by both environmental and human predictive factors. Human activities can directly or indirectly affect the process of biological invasion [69,70,71]. Human trade or tourism activities can break the geographical barriers in the process of species spread and lead to changes in the geographical distribution of organisms [72]. Environmental factors can affect how many species can survive in a new location, while human factors can affect the time, number, and ways of introducing species into the site [69,73,74]. Human influence can occur through propagule pressure (i.e., introduction number or introduction probability) [75] or the creation of a microclimate to allow organisms to survive in a climate that would otherwise be unsuitable [76]. Incorporating human factors into the climate niche model can improve the prediction of the niche model for areas with numerous human activities and can also obtain higher accuracy than models that only consider climate variables [77,78]. Among the three models constructed in this study, the human influence index (HII) is one of the most important predictors related to the distribution of *A. artemisiifolia* (Table 2). This is in line with the findings of Xian et al.’s study, which indicated that human activities are closely related to ragweed’s potential distribution [79]. If the large-scale climatic conditions are satisfied, biological interactions and human interference will become significant on smaller scales [78,80]. This may be because human activities help the long-distance spread of *A. artemisiifolia* seeds. Chapman et al. have shown that reproductive pressures such as human activity can accelerate the invasion of ragweed in Europe [81]. Human transportation routes (such as coastal waterways, highways, railways, and navigable rivers) promote the colonization of invasive species [82,83]. Additionally, anthropogenic alterations of the natural ecosystem through land use changes and infrastructure expansion (including construction, nocturnal lighting, and land cover changes) also encourage invasive colonization.

The limitations of the Maxent modeling software itself and the associated uncertainties should be an important aspect considered in this study [28]. While Maxent outperforms other similar niche models in general, it excels when dealing with small samples [84,85,86]. However, sample size, incomplete species occurrence data, failure to consider biological processes, selection of abiotic variables and spatial resolution, multicollinearity, and species characteristics may still have an impact on niche model predictions [28,87,88,89]. We only used the Maxent model in this study because we only had presence data and no available missing data. Although we increased the predictability and accuracy of the results by including reproductive pressure (represented by human activity effects) in the model construction, the results should be interpreted with caution due to the uncertainties inherent in Maxent and model-specific assumptions.

## 4. Methods

### 4.1. Species Occurrence Data

We collected a total of 41,296 global observations regarding *A. artemisiifolia* occurrences in both native and invasive areas from the Global Biodiversity Information Facility [90] and the Chinese Virtual Herbarium (CVH, https://www.cvh.ac.cn/, accessed on 12 February 2023). After deleting duplicated and incorrect data from locations in the ocean, we constructed a 5 km × 5 km grid to reduce the impact of sampling bias on ecological spaces and niche model prediction. The species distribution point nearest to the center of each grid was selected. A total of 15,731 sites were reserved for eco-spatial analysis and modeling. There were 9514 total invasion sites, including 49 in Africa, 335 in Australia, 412 in China, 7920 in Europe, and 140 in South America. In addition, 6217 sites were native to North America (Figure 5).

### 4.2. Environmental Data

The invasion process is driven by many biotic and abiotic factors. Abiotic environmental factors (such as climate factors) are key components of successful invasions [69,91]. We downloaded data pertaining to 19 bioclimatic factors and elevation data with a resolution of 2.5 arcminutes (~5 km^2^) from the WorldClim database [92]. Additionally, human activities can influence the course of non-native species invasion [69,70]. Therefore, we considered the influence of human factors on the distribution of *A. artemisiifolia* when selecting environmental variables. The human influence index with a resolution of 30 arc-second (~1 km^2^) from the Last of the Wild, v2 (http://sedac.ciesin.columbia.edu/, accessed on 27 July 2021), which represents humanity’s impact from 1995–2004, was used for modeling. Data on human population pressure (population density), anthropogenic land use and infrastructure expansion (construction, nocturnal lighting, and land cover changes), and human transportation routes (coastlines, roads, railroads, and navigable rivers) were obtained from the Wildlife Conservation Society [93] (Table 3).

First, we resampled the resolution of all environment variables to 2.5 arc minutes using ArcGIS 10.2’s resampling tool. ENMTools was used to calculate Pearson correlations between all variables in order to avoid model overfitting [94]. Then, we used the default parameters of the model for pre-modeling and deleted the variables with Pearson correlation coefficients greater than 0.8 while also removing variables with low contribution rates [95,96]. Finally, the model was constructed using temperature seasonality (Bio_4), annual precipitation (Bio_12), precipitation during the driest month (Bio_14), and the Human Influence Index (HII). We used principal component analysis (PCA-env) to measure and estimate the climatic niche of ragweed by selecting three climatic variables (Bio_4, Bio_12, and Bio_14) from four environmental variables.

### 4.3. Ecological Niche Modeling

Maxent (Version 3.4.3) [97] was used to simulate the realized niche and spatial distribution of *A. artemisiifolia*. Many studies have shown that Maxent has better stability and predictive performance than similar niche models [84,85,86]. We tested the predictive performance of the model with 25% of the location data. During the operation of the model, we set the maximum number of iterations as 5000 to attain a convergence threshold of 0.00001. Additionally, the feature combination (FC) and regularization multiplier (RM) were optimized. We combined 15 feature classes (including linearity (L), quadratic (Q), product (P), and hinge (H)) and 40 regularized multipliers using the R v3.6.3 program and the “kuenm” package [98] (from 0 to 4.0, with an interval of 0.1). A total of 600 candidate models were generated in the end. All models were evaluated with a 0.05 omission rate and a delta AICc (Akaike information criterion corrected for small sample size) less than 2 [98].

### 4.4. Climatic Niche Space Comparison: “COUE” Framework

We use the statistical framework developed by Broennimann [99] to quantify and compare the differences in climate and spatial environmental conditions between the native habitats and all the invaded areas. We used PCA-env ordination techniques to quantify the overlap of niches between different ranges during this process. This method combines environmental variables measured from all available sites at the scales of the native and invaded areas and projects them onto the first two principal component axes while also gridding the environmental spatial data and occurrence records. To directly compare the occurrence density between native and available habitats in the invaded areas, the density of each grid cell and available environment space was smoothed using a Gaussian kernel with a standard bandwidth [99]. Finally, we estimated the realized niche expansion, unfilling, and stability using the gridded environmental data and occurrence density records described above [21,33].

We used Schoener’s D [100] to quantify the degree of niche overlap between the native and invasive ragweed ranges. When D is 0, the niches in the two ranges have no overlap. When D is 1, the niches in the two ranges overlap completely. To determine whether the ecological niche of *A. artemisiifolia* has changed in the invaded range compared to that in its native range, we tested the niche equivalence and similarity between the two regions [101]. The niche equivalence test calculates the D value by combining all occurrences and randomly dividing them into the same initial number of native and invasive events. The niche similarity test simulates a niche by randomly shifting the entire observed occurrence density in one range and calculating its overlap with the observed niche in another range. The two processes were randomly repeated 100 times to construct the histogram of the simulated zero distribution of the D value [99,101]. The above comparative analysis of climate niche space was carried out in R using the “ecospat” package [102] and script developed by Broennimann et al. [99] (https://www.unil.ch/ecospat/en/home/menuguid/ecospat-resources/tools.html, accessed on 3 April 2021).

### 4.5. Invasion Stage Analysis in China

We constructed the model using the occurrence data of two ranges: (1) all occurrence data, excluding China, were used for a global joint model of origin and invasion (IN-Global); (2) all occurrence data in China were used to model the invasion of China (Inv-China). We used the theoretical framework suggested by Gallien et al. [103] to infer the current invasion stage of *A. artemisiifolia* in China by comparing the results of the joint global native (IN-global) and invasion region model with those of the invasion China model (Inv-China). If the invader population appears with high probability in the prediction results of “IN-global” and “Inv-China”, then it is a stable population. If the probability of occurrence of this population is low in the prediction results of “Inv-China” and high in the prediction results of “IN-global”, then the population is in a regional colonization state. If in the prediction results of “Inv-China” model, the probability of occurrence of this population is high, but in the “IN-global” model, the probability of occurrence of this population is low, and the population is in a regional adaptation stage. If the probability of occurrence of this population is low in the prediction results of the “IN-global” and “Inv-China” models, the population is in a sink state [103].

All GIS analyses were conducted using ArcGIS version 10.2.2 (Environmental Systems Research Institute: Redlands, CA, USA). Comparisons of niches in environmental space were conducted in R 4.1.3 (R Development Core Team 2013).

## 5. Conclusions

Our results reveal the climatic niche dynamics of ragweed during its invasion process. The native climatic niche of *A. artemisiifolia* was partially conserved as it spread to new regions throughout the globe. The climatic niches of most invasive populations represented a subset of the native climatic niches, suggesting that the invasion potential of non-native species cannot be entirely estimated by their distribution solely within the invaded region. Therefore, we suggest that both the native and invasive ranges of a species need to be considered when estimating the invasion potential of that species. The differences between the climatic niches in the invaded and native regions, excluding South America, are mainly due to climate-driven unfilling. The climatic niche unfilled in the invaded range is more likely due to dispersal limitations because biological invasions are a recent and ongoing process. *A. artemisiifolia* is better adapted to the low-temperature stress of the invaded region. The considerable differences in climatic conditions are the main reason for the expansion of the *A. artemisiifolia* climatic niche. In addition, through the ENM results, we found that there are many areas in China at risk of being invaded by ragweed, but these areas have not been invaded yet. Moreover, human activities play a substantial role in the southern expansion of *A. artemisiifolia*. We suggest that when considering the invasion potential of non-native species and assessing future risks, it is important to consider not only environmental factors, such as climate, but also how human activities may have affected the analyzed region.

Our results provide important information for ragweed management and control efforts. Our results from analyzing changes in the ecological niche of ragweed after its invasion demonstrate that there is still a significant potential for ragweed to invade. The different stages of ragweed invasion in China mapped in this paper can be used to prevent the future spread of ragweed as well as manage and monitor currently invaded areas. The analytical methods and theoretical framework used in this study are equally applicable to other harmful invasive species.

## Figures and Tables

**Table 1:** Niche overlap index (Schoener’s D), niche equivalence test and similarity test *p*-value, and niche dynamic index between the native range and invasive regions of *A. artemisiifolia*.

Invasive Region	Niche Overlap	Niche Equivalency	Niche Similarity		Unfilling	Stability	Expansion
	D	*p*-Value	Native > Invasive *p*-Value	Invasive > Native *p*-Value			
All Invasive	0.276	0.0099	0.0099	0.0099	0.276	0.992	0.008
Africa	0.045	0.0003	0.0396	0.0792	0.979	0.947	0.053
Australia	0.210	0.3267	0.0099	0.0099	0.964	0.975	0.025
China	0.122	0.0099	0.0198	0.0099	0.663	0.952	0.048
Europe	0.226	0.0099	0.0099	0.0099	0.559	0.999	0.001
South America	0.068	0.0099	0.0396	0.0495	0.846	0.593	0.407

**Table 2:** Average percent contribution and permutation importance of environmental variables to different *Ambrosia artemisiifolia* models; values were averaged across 10 replicate runs.

Environmental Variable	Native		Inv-China		IN-Global	
	Percent Contribution	Permutation Importance	Percent Contribution	Permutation Importance	Percent Contribution	Permutation Importance
Human influence index (HII)	35.8	37.8	43.1	32.9	40.3	46.1
Temperature seasonality (BIO_4, standard deviation × 100)	14.7	35.7	23.9	39.1	38.7	34.6
Annual precipitation (BIO_12, mm)	10.2	17.1	21.8	20	4.1	8.9
Precipitation of driest month (BIO_14, mm)	39.2	9.3	11.2	8	16.8	10.3

**Table 3:** Environmental variables for niche model building and climatic niche comparisons.

Variables	Description	Units
Bio_1	Annual mean temperature	°C
Bio_2	Mean diurnal range	°C
Bio_3	Isothermality	/
Bio_4	Temperature seasonality	/
Bio_5	Max temperature of warmest month	°C
Bio_6	Min temperature of coldest month	°C
Bio_7	Temperature annual range	°C
Bio_8	Mean temperature of wettest quarter	°C
Bio_9	Mean temperature of driest quarter	°C
Bio_10	Mean temperature of warmest quarter	°C
Bio_11	Mean temperature of coldest quarter	°C
Bio_12	Annual precipitation	mm
Bio_13	Precipitation of wettest month	mm
Bio_14	Precipitation of driest month	mm
Bio_15	Precipitation seasonality	/
Bio_16	Precipitation of wettest quarter	mm
Bio_17	Precipitation of driest quarter	mm
Bio_18	Precipitation of warmest quarter	mm
Bio_19	Precipitation of coldest quarter	mm
Alt	Elevation	m
HII	Human Influence Index	/

## Data Availability

Data will be made available on request.

## Supplementary Materials

The following supporting information can be downloaded at: https://www.mdpi.com/article/10.3390/plants12061313/s1, Figure S1: Multivariate environmental similarity surface analysis of native (North America) and invaded (South America) regions; Figure S2: Record sites of *Ambrosia artemisiifolia* occurrence in China.
