# Supplementary material for: Niche Filling Dynamics of Ragweed (Ambrosia artemisiifolia L.) during Global Invasion
Source: Plants (Basel). 2023 Mar 14;12(6):1313. doi: 10.3390/plants12061313 (PMC10055026; doi:10.3390/plants12061313)
Supplement: Supplementary file 1 [file plants-12-01313-s001.zip › plants-2248176-supplementary.pdf]

Supplementary Materials:

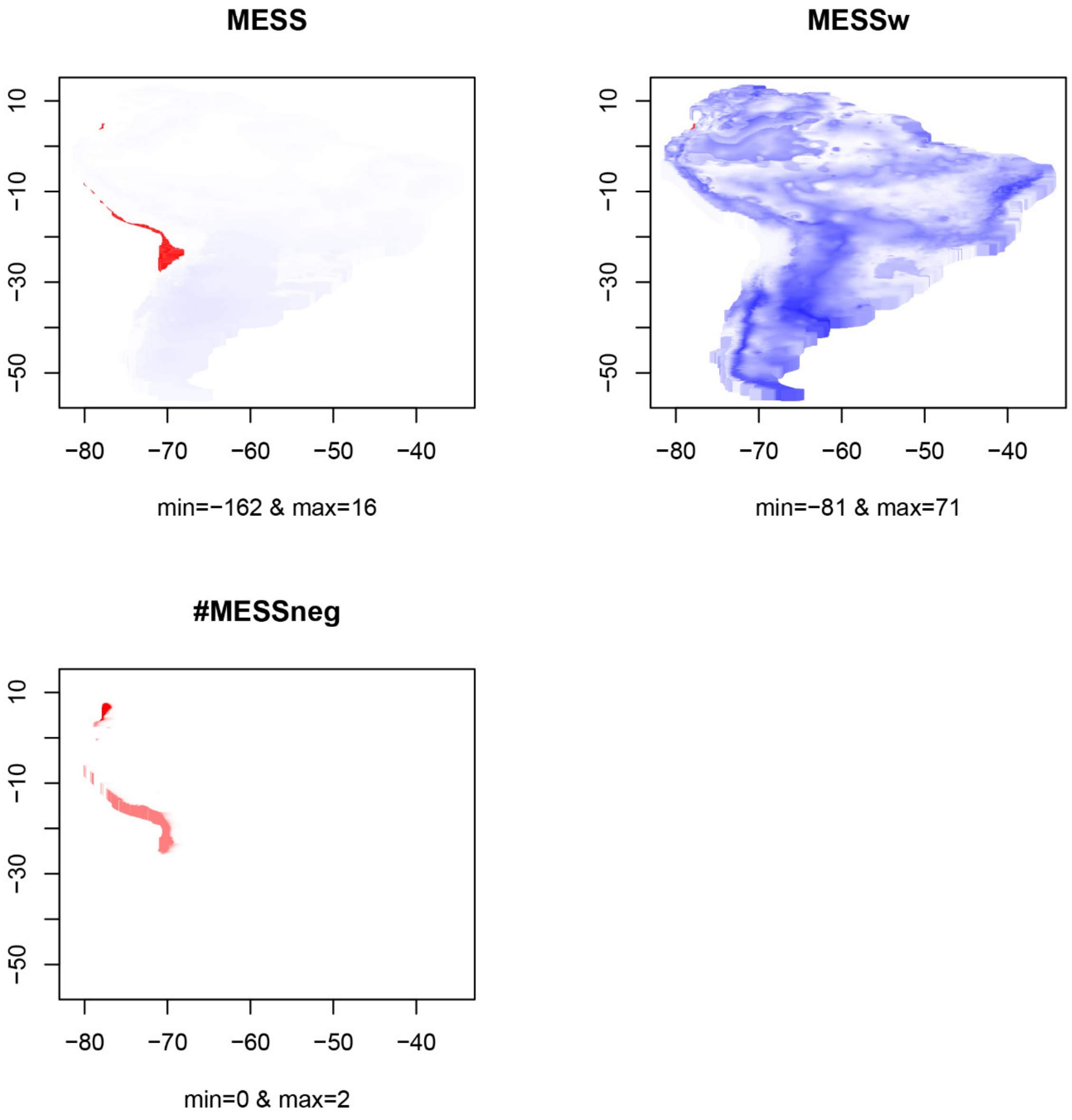

Figure S1 Multivariate environmental similarity surface analysis of native (North America) and invaded (South America) regions. Climatic conditions similar to the native niche are shown in red, and increasingly novel (different) climatic conditions relative to the native niche are shown in light blue to dark blue. MESS is the mess as calculated in Maxent, i.e. the minimal extrapolation values. MESSw is the sum of negative MESS values corrected by the total number of predictors. If there are no negative values, MESSw is the mean MESS. MESSneg is the number of predictors on which there is extrapolation.

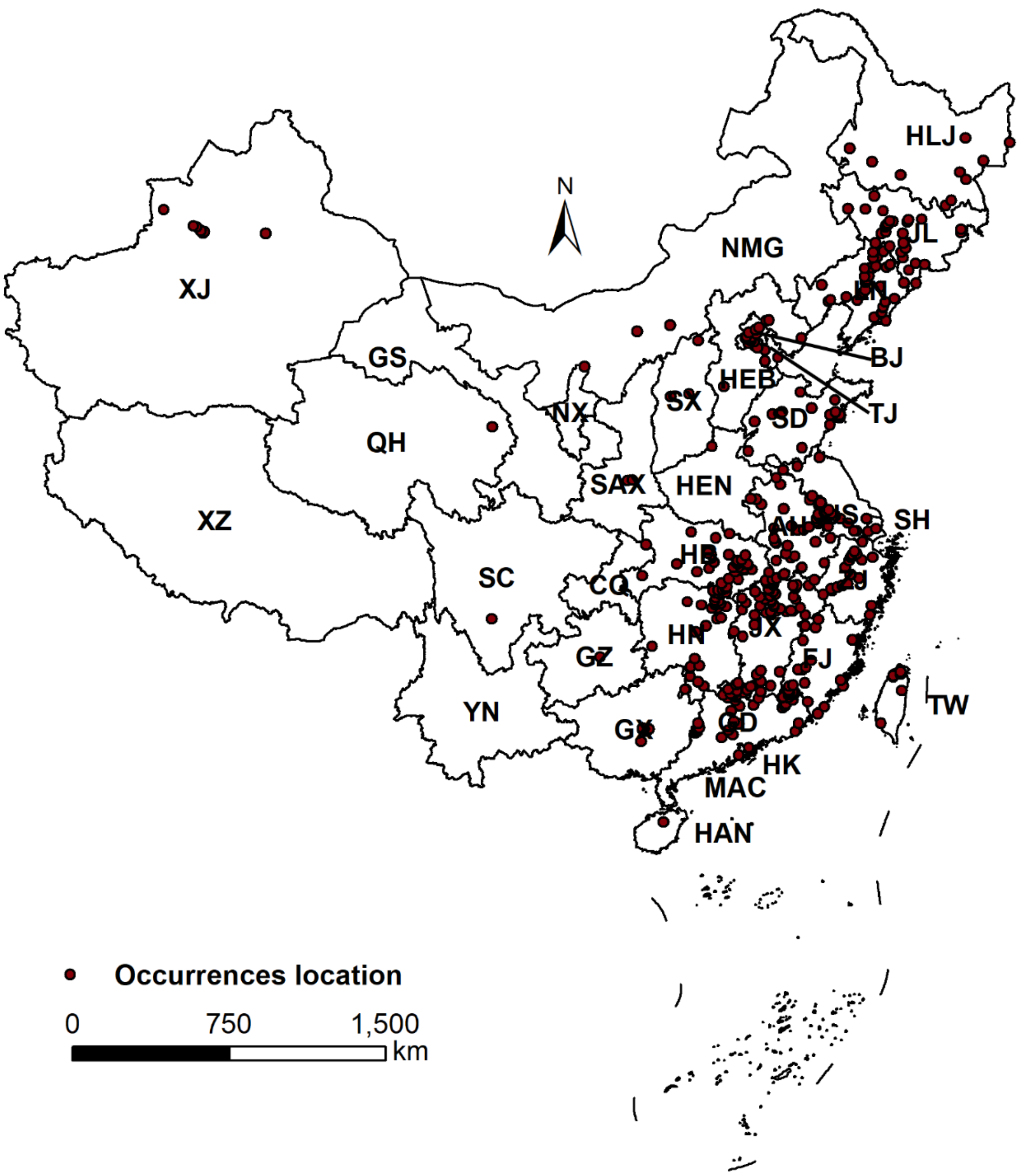

Figure S2 Record sites of *Ambrosia artemisiifolia* occurrence in China. Province abbreviations: AH = Anhui, BJ = Beijing, CQ = Chongqing, FJ = Fujian, GD = Guangdong, GS = Gansu, GX = Guangxi, GZ = Guizhou, HEN = Henan, HB = Hubei, HEB = Hebei, HAN = Hainan, HK = Hong Kong, HLJ = Heilongjiang, HN = Hunan, JL = Jilin, JS = Jiangsu, JX = Jiangxi, LN = Liaoning, MAC = Macau, NMG = Inner Mongolia, NX = Ningxia, QH = Qinghai, SC = Sichuan, SD = Shandong, SH = Shanghai, SAX = Shaanxi, SX = Shanxi, TJ = Tianjin, TW = Taiwan, XJ = Xinjiang, XZ = Tibet, YN = Yunnan, ZJ = Zhejiang.
